# Effects of Dietary Energy Density in a Fermented Total Mixed Ration Formulated with Different Ratios of Rice Straw and Cassava Pulp on 2- or 14-Day-Aged Meat Quality, Collagen, Fatty Acids, and Ribonucleotides of Native Thai Cattle Longissimus Muscle

**DOI:** 10.3390/foods11142046

**Published:** 2022-07-11

**Authors:** Chanporn Chaosap, Achara Lukkananukool, Sineenart Polyorach, Kritapon Sommart, Panneepa Sivapirunthep, Rutcharin Limsupavanich

**Affiliations:** 1 Department of Agricultural Education, School of Industrial Education and Technology, King Mongkut’s Institute of Technology Ladkrabang, Bangkok 10520, Thailand; panneepas@yahoo.com; 2 Department of Animal Production Technology and Fishery, School of Agricultural Technology, King Mongkut’s Institute of Technology Ladkrabang, Bangkok 10520, Thailand; achara.lu@kmitl.ac.th (A.L.); sineenart.po@kmitl.ac.th (S.P.); rutcharin.li@kmitl.ac.th (R.L.); 3 Department of Animal Science, Faculty of Agriculture, Khon Kaen University, Khon Kaen 40002, Thailand; kritapon@kku.ac.th

**Keywords:** beef, aging, collagen solubility, inosine monophosphate, meat flavor

## Abstract

This study investigated the effects of dietary energy density in rice straw and cassava pulp fermented total mixed ration on pH, cooking loss, Warner–Bratzler shear force (WBSF), and collagen content of 2- or 14-d-aged native Thai cattle (NTC) *Longissimus thoracic* (LT) muscles and fatty acids and ribonucleotides of 2-d-aged LT. Eighteen yearling NTC (*Bos indicus*) were randomly divided into three dietary treatments (T1 = 8.9, T2 = 9.7, and T3 = 10.5 MJ ME/kg), with six bulls per treatment. The results showed that T1 had the highest WBSF (*p* < 0.05). However, T2 had similar WBSF to T3 (*p* > 0.05). With aging, cooking loss increased (*p* < 0.01), while WBSF decreased (*p* < 0.01). Insoluble and total collagen decreased with aging (*p* < 0.05). Dietary energy density had no effect (*p* > 0.05) on collagen content, ribonucleotides and most fatty acids. However, T1 had more (*p* < 0.05) decanoic (C10:0), vaccenic (C18:1n9t), trans-linolelaidic (C18:2n6t), eicosatrienoic (C20:3n6), and docosadienoic (C22:2) acids than T2 and T3. In terms of lowest feed cost with comparable tenderness to T2 and highest energy density, T3 may be well suited for feeding NTC. Aging for 14 days improves LT tenderness, but its cooking loss may affect yield and juiciness.

## 1. Introduction

Beef consumption in Thailand is increasing, while the production is insufficient to meet the demand. Therefore, beef and beef-product-imported values of the country increased by 34% in 2021 compared to 2020 [1]. In 2020, 6.23 million cattle were raised in Thailand, of which 3.50 million or 56% were native Thai cattle (NTC) [2]. NTC, classified as *Bos indicus*, were formerly used as draught cattle in Thailand before being considered for meat production [3,4]. Despite their small size and slow growth, NTC are well adapted to the harsh and humid climate and resistant to ticks, parasites, and diseases [3,5]. They can utilize agricultural by-products or low-quality ingredients as feed and have high fecundity and maternal characteristics [3,5]. Farmers generally let them graze freely on low-quality local grasses and weeds without supplementation, except for some rice straw in the dry season. Depending on natural grazing alone results in low growth performance and low productivity [6]. Previous studies [6,7] suggested that NTC longissimus muscle offers health benefits due to its lower fat (<1.0%) and cholesterol content, but more polyunsaturated fatty acids (PUFAs), conjugated linoleic acid (CLA C18:2), eicosapentaenoic acid (EPA, C20:5n3), and docosapentaenoic acid (DHA, C22:5n3) than those of *Bos taurus* crosses. However, the meat from NTC was less tender with Warner–Bratzler shear force (WBSF) values of 10.47 kg [6] and 15.78 kg [4], which may be a limitation from an eating quality standpoint.

As consumers demand not only more quantity of meat, but also on a better quality, especially for eating quality, there is a need for NTC meat quality improvement. The feeding strategy is a tool to improve cattle performance, productivity, and meat quality. For example, concentrate-fed beef had more monounsaturated fatty acids (MUFA) in intramuscular fat, less connective tissue, and better palatability than grass-fed beef [8]. The high intramuscular fat content in concentrated-fed beef may improve the flavor, juiciness, and tenderness of the meat [8]. Fermented total mixed ration (FTMR) is a complete feed combination of concentrate and roughage, formulated to meet a specific nutrient requirement of cattle and fermented to provide better nutrient availability, feed intake, digestibility, and aerobic storability [9,10,11,12,13]. In Thailand, cassava (*Manihot esculenta* Crantz) is one of the most important agro-industrial crops with a production of about 31.6 million tons in 2021 [14]. It is mainly used as a raw material for the starch industry, producing a surplus of cassava pulp. Cassava pulp is composed of highly digestible starch and fiber, making it suitable as an alternative energy source for cattle feed [15,16,17], especially when compared to rice straw [17,18]. A study by [13] showed that inclusion of cassava pulp in TMR silage improved preserved nutritional value, feed storage ability, rumen fermentation, digestibility, and enteric methane mitigation in NTC. Higher growth performance of NTC fed with higher energy density by using more cassava pulp instead of rice straw in FTMR has been reported [12,17].

Post-mortem aging in a vacuum package or wet aging are known to be methods for improving meat palatability [19]. It is well recognized that post-mortem proteolysis of myofibrilla proteins is responsible for improving tenderness during aging [20,21,22]. With extended aging, proteolytic enzymes degrade and dissociate the intramuscular connective tissue structure (IMCT) of muscles with high connective tissue toughness, resulting in collagen solubilization [23]. The higher collagen content with lower collagen solubility causes meat toughness [24,25,26]. It has been known that both fatty acid composition and ribonucleotide content in meat contribute to meat flavor [27,28,29]. In addition, fatty acid composition in meat plays an important role in terms of the health aspect. MUFA are better for health, while saturated fatty acids (SFA) may cause health problems [30]. However, different SFAs have various biological effects, making their roles in heart disease complex [31].

This study was a continuation of the study of [32], which investigated the growth performance, carcass quality, and production cost of NTC fed with different energy densities and ratios of rice straw and cassava pulp FTMR. Therefore, the objective of the present study was to further investigate the effects of energy density in FTMR with different ratios of rice straw and cassava pulp on (1) meat quality, including pH, cooking loss, WBSF, and collagen solubility of 2- or 14-d post-mortem aged LT muscles of NTC and (2) fatty acid composition and ribonucleotide content of 2-d aged LT muscles of NTC.

## 2. Materials and Methods

### 2.1. Animal Ethics

The LT muscle samples used in this study were collected from 18 NTC bulls that were part of a government project supported by the Thailand Research Fund (project code RDG5820025). The cattle were raised at the Khon Kaen University Farm Research Station, Khon Kaen Province, Thailand. All experimental procedures were conducted in accordance with the animal welfare standards of the Animal Care and Use Committee of Department of Livestock Development, Ministry of Agriculture and Cooperatives, Royal Thai Government.

### 2.2. Experimental Cattle and Muscle Collection

Eighteen NTC bulls with an average age of 16.8 months and an average body weight of 96.1 ± 5.2 kg (mean ± SD) were randomly assigned to three treatment groups with different densities of metabolizable energy (ME) in FTMR: Treatment 1 (T1: 8.9 MJ ME/kg DM), Treatment 2 (T2: 9.7 MJ ME/kg DM), and Treatment 3 (T3: 10.5 MJ ME/kg DM), with six bulls per treatment. The experimental diet was formulated to meet the nutritional requirements of beef cattle [33] and to produce a total mixed ration (TMR) silage [17] using ingredients such as rice straw, cassava pulp, cassava chips, palm meal, soybean meal, rice bran, urea, and minerals. The ingredients and analyzed chemical composition of the FTMR formulation with different ratios of rice straw and cassava pulps are shown in Table 1 [32]. The diets were offered ad libitum as FTMRs to all cattle twice per day at 08:30 and 16:30. The experimental animals were randomly assigned to one of six blocks according to their initial body weight using a completely randomized block design. Within each block, animals were randomly allocated to one of the three dietary treatments and were fattened for 450 days. Animals were transported for approximately eight h to a commercial slaughterhouse where they were stalled for 12 h and had access to water prior to slaughter. The animals were weighed, stunned, bled, skinned, eviscerated, and washed according to commercial practices. Carcasses were split lengthwise and stored at 2 ± 2 °C for 24 h before transfer to the Meat Science and Technology Laboratory at King Mongkut’s Institute of Technology Ladkrabang, Bangkok, Thailand. At 2 days postmortem, the carcasses were fabricated. Then, LT muscle sample was taken from the left side of each carcass. All visible fat was removed from the samples, which was then divided into one 1.5 cm thick and two 5 cm thick portions. The 1.5 cm thick portion was vacuum packaged and stored at −80 °C for further analysis of ribonucleotide content and fatty acid composition. Each of the 5 cm thick portions was individually vacuum packaged in a barrier bag and stored at 1 °C for 2- or 14-day-aged meat quality analysis. For meat quality analysis, at each post mortem aging time, each 5 cm thick muscle portion was removed from a barrier bag and pH was measured. The muscle was then cut into 2 and 3 cm thick steaks, vacuum packaged individually in a barrier bag, and kept frozen at −20 °C. Each 2 cm thick steak was used for collagen content determination, while the 3 cm thick steak was used for cooking loss and WBSF measurements. 

### 2.3. Meat Quality Analysis

#### 2.3.1. pH Measurement

At 2 and 14 days postmortem, duplicate measurements of muscle pH were obtained using a pH meter equipped with a spear tip glass electrode (SevenGo, Mettler-Toledo International Inc., Greifensee, ZH, Switzerland).

#### 2.3.2. Cooking Loss and Shear Force Measurement

At each post-mortem aging time, the frozen vacuum packaged 3 cm thick steak was thawed overnight, removed from the bag, and weighed before being placed in a high-density polyethylene bag, heat sealed, and cooked in a water bath (80 °C) until the internal temperature reached 70 °C. The cooked samples were removed from the bag and allowed to cool to room temperature before being reweighed. The difference in weight between cooking was used to quantify cooking loss and expressed as a percentage of the weight before cooking. For WBSF measurements, each cooked sample was cut transversely and along the muscle fiber orientation into eight pieces of 1.3 × 1.3 × 3 cm^3^ cubes. Each cube was sheared perpendicular to the muscle fibers using the texture analyzer (EZ-SX, Shimadzu, Kyoto, Japan) equipped with a 50 kg load cell and a crosshead speed of 50 mm/min. The WBSF value of each muscle sample was calculated by averaging the values of eight cubes’ cuts.

#### 2.3.3. Collagen Measurement

According to [34], with slight modification, the 2 cm thick steak was homogenized with Ringer’s solution at 77 °C for 70 min and then centrifuged at 2500× *g* for 10 min. Soluble and insoluble collagen were measured by hydrolysis of the supernatant and sediment solutions in 12 N HCl and 6 N HCl at 110 °C for 20 h, respectively. Hydroxyproline concentration was determined by measuring the absorbance of the hydrolysate at 550 nm and comparing with a standard curve as described by [35]. Collagen content was determined by multiplying the hydroxyproline content by 7.25. 

#### 2.3.4. Ribonucleotide Measurement

The muscle sample obtained from the 1.5 cm thick 2-day-aged muscle portion was homogenized in 0.6 M perchloric acid as described by [36]. The homogenate was neutralized, pH adjusted to 7.0 with 0.8 M KOH and KH_2_PO_4_ buffer, and centrifuged at 10,000× *g* for 10 min at 4 °C. Inosine monophosphate (IMP), guanosine monophosphate (GMP), inosine, and hypoxanthine were determined in the supernatant. The stationary phase was a TSK gel Amide-80 column (Tosoh, Tokyo, Japan) and the eluent was a 70:30 acetonitrile:KH_2_PO_4_ buffer. Ribonucleotide content was determined using an external standard curve. 

#### 2.3.5. Fatty Acid Composition Measurement

Fatty acid composition was analyzed according to [37] using a muscle sample obtained from the 1.5 cm thick 2-day-aged muscle portion. Chloroform was used for the extraction of lipids according to the method of [38]. Methyl nonadecanoate was used as an internal standard during extraction. Fatty acid methyl esters (FAMEs) were analyzed by gas chromatography (7890B, Agilent, Santa Clara, CA, USA), using a fused silica capillary column (100 m × 0.25 mm × 0.2 μm film thickness, SPTM-2560, Supelco, Bellfonte, PA, USA). The peaks of FAMEs were detected and quantified by comparing the retention times with the standard of FAME C4–C24 components. The percentage of each fatty acid was calculated relative to the total fatty acid content.

### 2.4. Statistical Analysis

Data were analyzed with a general linear model using Proc GLM. Least-squares means were separated using the PDIFF option. Values of *p* < 0.05 were considered statistically significant. Statistical analysis was performed using SAS Institute Inc., Cary, NC, USA. For pH, cooking loss, shear force value, and collagen content, the experimental model included terms for dietary treatment and aging period according to the following equation: Y_ijk_ = µ + b_i_ + T_j_ + A_k_+ T_j_*A_k_ + €_ijk_, where Y_ijk_ was the dependent variable, µ was the overall mean, b_i_ was the block effect of initial body weight (i = 1,......, 6), T_j_ was the fixed effect of dietary energy density (j = 1, 2, 3), A_k_ was the fixed effect of the aging period (k = 1, 2), and T_j_ * A_k_ was interaction and €_ijk_ was the residual error. For ribonucleotides and fatty acid composition, the experimental model included a dietary treatment term according to the following equation: Y_ij_ = µ + b_i_ + T_j_ + €_ij_, where Y_ij_ was the dependent variable, µ was the overall mean, b_i_ was the block effect of initial body weight (i = 1,......, 6), T_j_ was the fixed effect of dietary energy density (j = 1, 2, 3), and €_ij_ was the residual error.

## 3. Results

### 3.1. Meat Quality

The effects of different dietary energy densities in FTMR and aging time on meat quality of LT muscle of NTC are shown in Table 2. There was no interaction (*p* > 0.05) between dietary energy density and aging time on muscle pH, cooking loss, and WBSF. The highest dietary-energy-density treatments, T3, tended to have a higher percentage of cooking loss than others (*p* = 0.051). The dietary energy density affected the WBSF value, as the high dietary energy density, 9.7 MJ ME/kg (T2), and 10.5 MJ ME/kg (T3), had a lower WBSF value (*p* < 0.05) than the low dietary energy density, 8.9 MJ ME/kg (T1). The longer aging of 14 days had a higher percentage of cooking losses but a lower WBSF value (*p* < 0.01) than the aging of 2 days.

Muscle pH influences many meat quality characteristics, including water-holding capacity, color lightness, tenderness, and shelf life [39]. After slaughter, muscle pH decreases in relation to the increase in lactic acid accumulation due to anaerobic glycolysis [40]. Table 2 shows that neither dietary energy density nor aging time affected muscle pH (*p* > 0.05). In contrast to our study, [41] reported lower ultimate muscle pH in F1 Angus × Chinese Xiangxi Yellow cattle fed a high-energy diet than in cattle fed a low-energy diet. The authors of [41] stated that the lower pH in their study was due to higher glycogen availability because of feeding a high-energy diet. The difference between our results and their report could be due to differences in cattle breed, age, or diet. Previous investigation in the same group of experimental cattle as in this study showed that growth parameters, especially average daily gain (ADG), did not differ at different energy densities [32]. At the final phase of fattening, NTC might reach a mature body weight resulting in no further weight gained [32]. The limited nutrient utilization of NTC might lead to similar muscle glycogen levels; therefore, muscle pH did not differ in the current study. However, [42] stated that muscle glycogen in an animal before slaughter is generally less affected by nutrition, compared to stress or muscle energy requirement before slaughter. According to [43], the pH values of LT muscles in NTC fattened at all dietary energy densities in this study were in the normal range, indicating optimal pre-slaughter handlings and meat quality. 

The cooking loss of meat is the loss of water during a cooking process. It reflects the ability of meat to retain water during cooking at high temperature and correlates negatively with meat juiciness [44]. For the meat processing industry, high cooking loss means lower meat yield, affects the appearance of the meat, and is, therefore, an important economic indicator [45]. Table 2 shows that percent cooking loss tends to be affected (*p* = 0.051) by the dietary energy density of FTMR. The LT muscles of NTC fed the highest energy density (T3) tended to have (*p* = 0.051) higher cooking loss percentage than the others. However, in the study of [41], the dietary energy density showed no effect on cooking loss. They reported that the average cooking loss was 31.5%, which was higher than the present study. 

Aging time had an effect (*p* < 0.05, Table 2) on the percentage of cooking losses. LT muscles that were aged longer (14 days) had a higher (*p* < 0.05) percentage of cooking losses than muscles that were aged for only 2 days. The authors of [46] reported that 14-day-aged muscles had a 3% higher cooking loss than non-aged muscles. In contrast, [47] found that aging for 2 and 14 days had no effect on cooking loss and the average was 21.8%. The authors of [44] reported that cooking losses were lower for beef aged on day 0 than on day 7 (*p* < 0.05), with averages of 25.8% and 30.7%, respectively. The higher cooking loss during aging may be due to the denaturation of muscle protein and degradation of certain muscle proteins, such as desmin and troponin T, by postmortem proteolysis, resulting in a decrease in water-holding capacity in aged beef [20,48]. In addition, it has been shown that in aged beef, water tends to shift from the intracellular to the extracellular compartment, making it easier to remove during cooking [46,49].

WBSF assessment is an objective measurement of a force required to cut through muscle fiber bundles simulating a biting process. A higher WBSF indicates less tenderness. Meat was defined as “tender” when it had a shear force value of less than 6 kg [50]. Results showed that both dietary energy density in FTMR and aging time influenced WBSF values (*p* < 0.05, Table 2). The LT muscles of NTC in the high-dietary-energy-density treatments (T2, 9.7 MJ/kg and T3, 10.5 MJ/kg) resulted in a lower WBSF value (*p* < 0.05) than in the lowest-dietary-energy-density treatment (T1, 8.9 MJ/kg). The result of the present study is consistent with [41,51]. A study by [51] showed that the lower shear force value was associated with a higher muscle fragmentation index in cattle fed high-energy diets than in cattle fed low-energy diets. The possible explanation for the lower WBSF value in the high-energy FTMR (T3) in the current study could be due to slightly more (*p* < 0.05) marbling accumulation in T3, as reported in the study by [32], which conducted a study on the same group of experimental cattle as this study. In addition, [32] reported the fat percentages of the LT muscles as 6.1, 6.2, 6.7 for T1, T2, and T3, respectively (*p* > 0.05). According to [52], in Holstein-Friesian bulls, increased dietary energy, with less structural carbohydrate, resulted in an increase in ruminal amylolytic bacteria, but decreasing cellulolytic bacteria. This further increased propionate, but reduced acetate concentration, influencing blood glucose and allowing more carbon source as a substrate for intramuscular lipogenesis [52,53]. As shown in Table 1, T3 has the highest proportion of cassava pulp, but the least neutral detergent fiber (NDF) and acid detergent fiber (ADF). However, [32] found no significant difference (*p* > 0.05) between the marbling score (MS) of T1 and T2. Thus, MS may not be the only factor responsible for the tenderness of the LT muscle in this study. As expected, LT exhibited lower WBSF at 14 days of aging (*p* < 0.01) than at 2 days of aging. 

### 3.2. Collagen Content

Collagen is a major component in the connective tissue of meat. The higher the collagen content, the lower the solubility of collagen, or the stronger its structural cross-linking, resulting in higher toughness in meat [24,25,26]. Table 3 shows the effects of dietary energy density in FTMR and aging time on collagen parameters in LT muscles of NTC. There was no combined effect (*p* > 0.05, Table 3) of dietary energy density and aging time on any collagen parameters. There was a trend (*p* < 0.1) toward higher insoluble and total collagen content in the medium energy FTMR (T2; rice straw 30: cassava pulp 30) than in the others. However, there was no effect of energy density on soluble collagen and percent collagen solubility. In contrast, higher collagen solubility was found in the muscles of cattle fed a high-energy-density diet than in cattle fed a low-energy-density diet [51]. In another study, soluble collagen concentration was higher in cattle fed high-energy diets (*p* < 0.084), whereas dietary energy density had no effect on total collagen content [28]. The authors of [54] noted that collagen solubility increased in the longissimus muscle of grain-fed cattle, which was probably due to an increase in collagen biosynthesis rate or a decrease in collagen cross-linking rate after the animals were fed a higher-dietary-energy-density diet. The explanation for the nonsignificant differences in collagen solubility between treatments in the current study may be related to the limited nutrient utilization of NTC, as indicated by the nonvariation in ADG at different dietary energy densities, as mentioned in the report of [32]. However, the higher proportion of insoluble collagen and total collagen in the treatment, which contained a similar proportion of rice straw and cassava pulp, may require further investigation. 

Table 3 shows that post-mortem aging time had an effect (*p* < 0.05) on insoluble collagen content and total collagen content in LT muscles. The 14-day-aged LT muscle had less (*p* < 0.05) insoluble and total collagen than that aged after 2 days. The decrease in these two collagen parameters may indicate post-mortem degradation of collagen structure, which could be a contributing factor to the observed lower 14-day-aged WBSF value. However, soluble collagen and collagen solubility percentage were not affected by aging time (*p* > 0.05, Table 3). The effect of aging on collagen solubility is controversial, as [44] found that post-mortem aging does not affect collagen content, while [55] reported that collagen is damaged and partially solubilized during aging.

### 3.3. Fatty Acid Composition

Fatty acid deposition in beef muscle is dependent, but not proportionately, on dietary fatty acid composition and rumen microbial dehydrogenation activity, which transforms UFA to SFA [56]. In general, MUFAs and PUFAs are considered better for health, while the image of SFAs is unhealthy for consumers [30]. However, high content of PUFAs in muscle foods can lead to oxidative rancidity, affecting meat flavor, storage ability, and overall meat quality [57]. Table 4 shows the effect of dietary energy density in FTMR with different ratios of rice straw and cassava pulp on fatty acid composition of LT muscle of NTC at 2-day aging. For most fatty acid compositions in LT muscle, the dietary energy density of FTMR showed similar effects (*p* > 0.05). However, dietary energy density affected (*p* < 0.05) decanoic acid (C10:0), vaccenic acid (C18:1n9t), trans-linolelaidic acid (C18:2n6t), eicosatrienoic acid (C20:3n6), and docosadienoic acid (C22:2) content in LT muscle. NTC fed the lowest-energy-density diet (T1, 8.9 MJ ME/kg) had higher (*p* < 0.05) levels of decanoic acid (C10:0), vaccenic acid (C18:1n9t), trans-linolelaidic acid (C18:2n6t), eicosatrienoic acid (C20:3n6), and docosadienoic acid (C22:2), and tended to have (*p* = 0.082) more linoleic acid (C18:2n6c), an essential fatty acid, than T2 (9.7 MJ ME/kg) and T3 (10.5 MJ ME/kg). However, fatty acid composition in T2 was similar to T3 (*p* > 0.05). This could be due to the influence of different ruminal fibrolytic bacterial communities predominated in T1, compared to T2 and T3 [17]. T1 contained a higher proportion of rice straw (50) to cassava pulp (10) ratio, which resulted in more dietary fiber in NDF and ADF (Table 1). According to [17,58], rumen microbial community was mainly affected by dietary forage to concentrate ratio, rather than dietary energy level, provided the energy level difference was at 8%. The three dietary-energy-density treatments, however, showed similar results (*p* > 0.05, Table 4) for SFA, MUFA, PUFA, and UFA (unsaturated fatty acids) content, as well as for UFA: SFA and PUFA:SFA ratios. It should be noted that MUFA was the most abundant fatty acid (65.74%), followed by SFA (31.33%) and PUFA (2.93%). 

The three most abundant fatty acids found in LT muscle examined in this study were oleic acid (C18:1n9c; 58.28–61.75%), palmitic acid (C16:0; 18.35–20.08%), and stearic acid (C18:0; 6.41–7.95%), respectively. These results agree with other studies [59,60,61]. Oleic acid, a MUFA, is abundant in grain-fed beef, which could be considered an important dietary source of MUFAs for humans [62]. Higher amounts of oleic acid in beef may be beneficial because it can increase blood HDL cholesterol levels and reduce the risk of cardiovascular disease (CVD) [30]. Consumption of trans fatty acids (TFA) has been associated with a number of health harms, particularly coronary heart disease (CHD), CVD, and related conditions [63]. The main sources of TFA are products from industrially produced hydrogenated vegetable oils, such as margarine and deep-fried foods, and a small amount of TFA may occur naturally in animal fat synthesized by anaerobic bacteria in the rumen [64]. In the present study, the highest amount of TFA (vaccenic acid 0.52% and trans-linolelaidic acid 0.49%) was found in T1, which accounted for only 1.01% of the total fatty acid (0.04 g/100 g), suggesting that the TFA in LT muscle from NTC in the present study contained less TFA than the recommended threshold of 5 g per 100 g serving, which is the threshold for increased CHD risk [64]. In ruminants, however, vaccenic acid is a substrate for the formation of CLA (C18:2 cis-9 tran-11) in muscle tissues [65,66]. In addition, a study in an animal model indicated that vaccenic acid-rich butter could prevent atherosclerosis development [67]. The authors [68] reported that medium-chain fatty acids (MCFAs) with 6–12 carbons have a unique transport system and are rapidly metabolized in the body. MCFAs not only serve as an energy source, but also regulate glucose and lipid metabolism. MCFAs have been found to enhance immune response and insulin secretion and may increase apoptosis in cancer cells. Therefore, the higher proportion of MCFA decanoic acid (C10:0) in T1 may contribute to some health benefits.

### 3.4. Ribonucleotides

Post-mortem degradation of nucleotide triphosphates, such as adenosine triphosphate (ATP) and guanosine triphosphate (GTP), results in flavor-related compounds, such as IMP and GMP [27,29]. IMP is reported to be responsible for umami flavor, while hypoxanthine is associated with a bitter taste, but inosine is tasteless [27,29]. From Table 5, dietary energy density in FTMR had no effect (*p* > 0.05) on the levels of hypoxanthine, inosine, IMP, and GMP in 2-day-aged LT muscles from NTC. This indicated that the three dietary energy densities of FTMR formulated by replacing rice straw with cassava pulp in this study resulted in similar ribonucleotide contents in 2-day-aged LT muscles of NTC. IMP was present in the highest amount, followed by inosine, hypoxanthine, and GMP, which ranged from 211.29–219.04, 19.85–21.41, 4.21–5.31, and 2.78–3.04 mg/100g, respectively (Table 5).

The fact that dietary energy density did not affect ribonucleotide content in the current study could also be related to the limitation in nutrient utilization of NTC [32], as mentioned earlier. Our study is in agreement with [69], who investigated the effects of carbohydrate sources (corn silage, corn grain, and barley grain) on meat quality of Xiangxi yellow cattle. They found that despite the lower ME in the corn silage group, the content of IMP and GMP was not affected compared to the higher ME corn grain and barley grain groups. However, [59] found that starch source affected IMP content, as pineapple stem starch had higher levels of IMP than ground corn and cassava starch in the LT muscle of Holstein steers. 

## 4. Conclusions

The three dietary-energy-density treatments did not affect pH, collagen, ribonucleotides, and most fatty acid parameters in LT muscles. The high-energy FTMR (rice straw 10: cassava pulp 50) can be considered for feeding NTC because it is the most economical diet, while it provides similarly tender LT as that of T2. Vacuum aging for 14 days, with careful handlings to prevent cooking loss, can be practiced for tenderness improvement. Further sensory quality study could provide more information on quality and consumer acceptance. Rumen microbial structure investigation in the low-energy-density FTMR (rice straw 50: cassava pulp 10) fed NTC may provide more understanding on its muscle fatty acid composition, some of which might have health benefits. It should be noted, however, that the most abundant fatty acid obtained in this study was MUFA, especially oleic acid (18:1n-9c).

## Figures and Tables

**Table 1:** Ingredients and chemical composition of dietary-energy-density treatment in native Thai cattle diet [32].

Item	Dietary Treatment ^1^			Cassava Pulp	Rice Straw
	T1	T2	T3		
Ingredients, %DM					
Rice straw	50	30	10		
Cassava pulp	10	30	50		
Cassava chip	7	7	7		
Palm meal	12	12	12		
Soybean meal	14	14	14		
Rice bran	5	5	5		
Urea	0.7	0.7	0.7		
Mineral ^2^	0.8	0.8	0.8		
Limestone	0.5	0.5	0.5		
Total	100	100	100		
Analyzed chemical composition, %DM					
Dry matter	46.2	41.1	33.2	23.8	92.8
Organic matter	88.4	90.8	93.6	98.2	88.0
Crude protein	13.8	14.3	15.0	2.2	2.7
Ether extract	3.5	3.3	3.4	0.8	0.9
Neutral detergent fiber	51.1	44.3	38.1	32.8	77.0
Acid detergent fiber	36.8	34.0	25.4	20.0	51.6
Energy density, MJ/kg DM					
Gross energy	17.0	17.4	17.6	16.4	16.2
Metabolizable energy (calculated)	8.9	9.7	10.5	9.6	6.5
Cost, Baht/kg FM	4.96	4.74	3.95	0.42	2.31
Cost, Baht/kg DM	13.19	12.61	10.51	1.74	2.49

^1^ T1 = 8.9, T2 = 9.7, T3 = 10.5 MJ ME/kg DM, ME = Metabolizable energy density. ^2^ Chemical compositions were calcium = 164.00 g, cobalt = 0.04 g, copper = 1.00 g, iodine = 0.04 g, iron = 2.00 g, magnesium = 2.89 g, manganese = 11.00 g, phosphorus = 80.00 g, selenium = 0.03 g, sodium = 136.60 g, sulfur =19.20 g and carrier = 1000.00 g.

**Table 2:** Effect of dietary-energy-density treatment (T) ^1^ and aging time (A) on pH, cooking loss, and Warner–Bratzler shear force (WBSF) of *Longissimus thoracis* muscles of native Thai cattle.

Trait	Treatment (T) ^1^			Aging (A)		RMSE ^2^	*p*-Value		
	T1	T2	T3	2 Days	14 Days		T	A	T*As
pH	5.65	5.56	5.59	5.56	5.63	0.18	0.543	0.349	0.969
Cooking loss (%)	20.81	20.78	23.87	19.95 ^b^	23.68 ^a^	2.91	0.051	0.002	0.066
WBSF (kg)	8.91 ^a^	7.26 ^b^	7.74 ^b^	9.40 ^a^	6.34 ^b^	1.42	0.024	<0.0001	0.802

^a,b^ LS means having different superscripts within the same main effect are different (*p* < 0.05). ^1^ T1 = 8.9 MJ ME/kg, T2 = 9.7 MJ ME/kg, T3 = 10.5 MJ ME/kg; ^2^ root mean square error.

**Table 3:** Effect of dietary-energy-density treatment (T) ^1^ and aging time (A) on soluble, insoluble, and total collagen contents (mg/g wet weight) and collagen solubility (%) in *Longissimus thoracis* muscle of native Thai cattle.

Collagen	Treatment (T) ^1^			Aging Time		RMSE ^2^	*p*-Value		
	T1	T2	T3	2 Days	14 Days		T	A	T*A
Soluble	0.17	0.20	0.18	0.18	0.19	0.06	0.537	0.372	0.574
Insoluble	2.58	3.00	2.76	2.96 ^a^	2.61 ^b^	0.42	0.095	0.023	0.468
Total	2.75	3.20	2.95	3.14 ^a^	2.80 ^b^	0.43	0.077	0.037	0.462
% solubility	6.32	6.34	6.32	5.68	6.97	2.19	0.999	0.110	0.585

^a,b^ LS means with different superscripts within the same main effect are different (*p* < 0.05). ^1^ T1 = 8.9 MJ ME/kg, T2 = 9.7 MJ ME/kg, T3 = 10.5 MJ ME/kg; ^2^ root mean square error.

**Table 4:** Effect of dietary-energy-density treatment (T) ^1^ on fatty acid composition of *Longissimus thoracis* muscle of native Thai cattle aged for 2 days.

Trait	Treatment ^1^			RMSE ^2^	*p*-Value
	T1	T2	T3		
Fatty acid composition	g/100 g total fatty acids (mg/100 g fresh meat)				
Decanoic acid (C10:0)	0.33 ^a^ (14.82)	0.12 ^b^ (7.79)	0.14 ^b^ (5.46)	0.11	0.024
Lauric acid (C12:0)	0.11 (5.01)	0.11 (5.99)	0.08 (3.26)	0.05	0.549
Tridecanoic acid (C13:0)	0.07 (2.77)	0.06 (3.69)	0.06 (2.53)	0.03	0.987
Myristic acid (C14:0)	2.73 (116.90)	3.02 (174.41)	2.52 (107.25)	0.76	0.636
Myristoleic acid (C14:1)	0.89 (38.82)	1.10 (62.70)	1.18 (49.60)	0.36	0.449
Pentadecanoic acid (C15:0)	0.26 (10.71)	0.19 (10.86)	0.20 (8.45)	0.06	0.162
Ginkgolic acid (C15:1)	0.12 (5.60)	0.10 (6.21)	0.13 (5.13)	0.03	0.510
Palmitic acid (C16:0)	20.08 (836.20)	20.67 (1209.13)	18.35 (789.84)	1.83	0.201
Palmitoleic acid (C16:1)	3.31 (140.01)	3.73 (215.27)	3.98 (169.45)	0.52	0.160
Margaric acid (C17:0)	0.69 (29.99)	0.70 (39.99)	0.69 (29.90)	0.10	0.998
Heptadecenoic acid (C17:1)	0.51 (21.97)	0.51 (29.23)	0.62 (26.37)	0.08	0.139
Stearic acid (C18:0)	7.95 (328.81)	7.21 (424.02)	6.41 (275.35)	1.50	0.310
Vaccenic acid (C18:1n9t)	0.52 ^a^ (21.75)	0.15 ^b^ (8.82)	0.24 ^b^ (10.18)	0.12	0.003
Oleic acid (C18:1n9c)	58.28 (2444.18)	59.27 (3443.02)	61.75 (2710.62)	4.58	0.498
Trans-Linolelaidic acid (C18:2n6t)	0.49 ^a^ (20.50)	0.20 ^b^ (11.49)	0.20 ^b^ (8.09)	0.15	0.022
Linoleic acid (C18:2n6c)	1.44 (57.41)	0.80 (46.81)	0.92 (37.25)	0.43	0.082
γ-Linolenic acid (C18:3n6)	0.14 (6.01)	0.66 (40.80)	0.75 (27.58)	0.49	0.148
∝-Linolenic acid (C18:3n3)	0.03 (1.61)	0.04 (2.24)	0.04 (1.64)	0.01	0.947
Arachidic (C20:0)	0.19 (8.07)	0.17 (10.24)	0.17 (7.64)	0.04	0.498
Erucic acid (C20:1n9)	0.18 (7.37)	0.15 (8.69)	0.17 (7.06)	0.03	0.502
Heneicosanoic Acid (C21:0)	0.12 (4.87)	0.14 (8.52)	0.17 (8.06)	0.04	0.116
Eicosatrienoic acid (C20:3n6)	0.31 ^a^ (13.09)	0.09 ^b^ (5.23)	0.18 ^b^ (6.63)	0.10	0.022
Docosanoic Acid (C22:0)	0.10 (3.96)	0.06 (4.06)	0.09 (3.47)	0.05	0.623
Eicosatrienoic acid (C20:3n3) + Arachidonic acid (C20:4n6)	0.55 (22.16)	0.31 (18.61)	0.41 (15.52)	0.23	0.295
Docosadienoic acid (C22:2)	0.07 ^a^ (2.97)	0.03 ^b^ (1.87)	0.04 ^b^ (1.30)	0.02	0.044
Nervonic acid (C24:1)	0.13 (5.52)	0.08 (4.70)	0.09 (3.48)	0.05	0.176
Docosahexaenoic acid (C22:6n3)	0.37 (15.77)	0.30 (18.22)	0.41 (17.35)	0.09	0.274
Total fatty acid (g/100 g)	4.19	5.82	4.34	1.38	0.188
SFA (saturated fatty acid)	32.63	32.46	28.90	3.77	0.282
MUFA (monounsaturated fatty acid)	63.96	65.11	68.15	4.49	0.359
PUFA (polyunsaturated fatty acid)	3.41	2.43	2.95	1.06	0.369
UFA (unsaturated fatty acid)	78.73	77.17	80.45	3.77	0.282
UFA:SFA	2.12	2.11	2.46	0.37	0.326
PUFA:SFA	0.10	0.07	0.10	0.03	0.274

^a,b^ LS means having different superscripts in the same row are different (*p* < 0.05).; ^1^ T1 = 8.9 MJ ME/kg, T2 = 9.7 MJ ME/kg, T3 = 10.5 MJ ME/kg; ^2^ root mean square error; SFA= C10:0 + C12:0 + C13:0 + C14:0 + C15:0 + C16:0 + C17:0 + C18:0 + C20:0 + C21:0 + C22:0; MUFA = C14:1 + C15:1 + C16:1 + C17:1 + C18:1n9t + C18:1n9c + 20:1n9 + C24:1; PUFA = C18:2n6t + C18:2n6c + C18:3n6 + C18:3n3 + C20:3n6 + (C20:3n3+C20:4n6) + C22:2 + C22:6n3; UFA = MUFA + PUFA.

**Table 5:** Effect of dietary-energy-density treatment (T) ^1^ on ribonucleotide content of *Longissimus thoracis* muscle of native Thai cattle aged for 2 days.

Ribonucleotides (mg/100 g)	Treatment ^1^			RMSE ^2^	*p*-Value
	T1	T2	T3		
Hypoxanthine	5.04	4.21	5.31	1.17	0.424
Inosine	19.85	21.08	21.41	6.69	0.927
Inosine monophosphate	219.04	211.29	213.95	15.07	0.707
Guanosine monophosphate	2.86	2.78	3.04	0.20	0.277

^1^ T1 = 8.9 MJ ME/kg, T2 = 9.7 MJ ME/kg, T3 = 10.5 MJ ME/kg; ^2^ root mean square error.

## Data Availability

The datasets generated during and/or analyzed during the current study are available from the author C.C. on reasonable request.
